# The Roles of Porous Coral Sands in Initial Enrichment of Ammonia-Oxidizing Bacteria

**DOI:** 10.1100/tsw.2007.108

**Published:** 2007-04-30

**Authors:** Qing Guo, Zao-he Wu, Ming-liang Qian, Binhe Gu

**Affiliations:** ^1^ South China Sea Institute of Oceanography, Chinese Academy of Sciences, 164 Xingangxi Road, Haizhu District, Guangzhou 510301, China; ^2^ Graduate School of Chinese Academy of Sciences, Beijing 100049, China; ^3^ Key Laboratory of Pathogen Biology and Epidemiology of Aquatic Economic Animals of Guangdong Province, Guangdong Ocean University, 40 East Jiefang Road, Xiashan District, Zhanjiang 524025, China; ^4^ Fishery College, Guangdong Ocean University, 40 East Jiefang Road, Xiashan District, Zhanjiang 524025, China; ^5^ Everglades Division, South Florida Water Management District, 3301 Gun Club Road, West Palm Beach, FL 33406, USA

**Keywords:** ammonia-oxidizing bacteria (AOB), calcium source, coral sands, enrichment media, nutrients, periphytic, substrate

## Abstract

The purpose of this study was to investigate the roles of coral sands in the enrichment and isolation of ammonium-oxidizing bacteria (AOB). We hypothesized that the porous coral sands provided additional surface area and nutrients for the growth of periphytic AOB. In the present study, an orthogonal test was designed to compare the AOB conversion rates of ammonium-nitrogen (NH_4_^+^N) to nitrite-nitrogen (NO_2_^-^-N) among various combinations of culture media. Results showed that the conversion of NH_4_^+^N to NO_2_^-^-N increased significantly when the coral sands were added, implying that coral sands were beneficial to the growth of AOB. Additions of potassium dihydrogen phosphate (KH_2_PO_4_) or sodium bicarbonate (NaHCO_3_) to the media became unnecessary when coral sands were used, but the addition of KH_2_PO_4_ was needed when the molar nitrogen to phosphorus (N:P) ratio reached 10 in the enrichment media using calcium carbonate (CaCO_3_) powder as a calcium source.

## INTRODUCTION

Nitrifying bacteria play an important role in the nitrogen cycle in natural waters and wastewater treatment[1,2,3]. Nitrifying bacteria include two distinct physiological groups: ammonia-oxidizing bacteria (AOB) and nitrite-oxidizing bacteria[4,5]. Ammonium can be converted to nitrate by two successive oxidizing processes via these microbes[2,6].

Since Winogradsky isolated autotrophic nitrifying bacteria in pure culture in 1890, numerous studies have been conducted on the enrichment, isolation, purification, and application of nitrifying bacteria[7,8]. Natural or artificial porous materials, such as ceramics, can be used as substrates for the growth of nitrifying bacteria, improving significantly their efficiency of converting ammonium-nitrogen ($NH_4^+$-N) to nitrite-nitrogen ($NO_2^-$-N) and $NO_2^-$-N to nitrate-nitrogen ($NO_3^-$-N). However, few studies have used porous materials in the initial enrichment of nitrifying bacteria[9,10]. In this study, porous coral sands containing mainly calcium carbonate ($CaCO_3$) and other chemical compounds were used to replace $CaCO_3$ powder, one of the commonly used calcium sources in initial enrichment media of AOB[2]. The differences in nitrification rate, i.e., the conversion of $NH_4^+$-N to $NO_2^-$-N via AOB, were compared when coral sands or $CaCO_3$ powder was used. In addition, the requirements for potassium dihydrogen phosphate ($KH_2PO_4$) and sodium bicarbonate ($NaHCO_3$) in culturing AOB were investigated.

## MATERIALS AND METHODS

### Materials

A detailed protocol of initial AOB enrichment and isolation has been reported elsewhere[11]. Briefly, AOB were enriched initially from a marine fish aquaculture system in southern China using a coral sands–containing liquid medium similar to that of Experiment No. 2 reported here. Seven rounds of transfer enrichment were conducted before the inoculum was used in this study. This enriched culture was used as an inoculum in all of the subsequent tests with an average density of AOB of approximately $5.8 \times 10^4$ cells $ml^{-1}$.

The coral sands, purchased at a local market for filter materials, were of various shapes with approximate volumes ranging from 60–120 $mm^3$. To remove organic matter from the surface, coral sands were soaked in diluted hydrochloric acid, washed copiously with tap water, boiled in distilled water, and dried. These pretreated coral sands were placed at the bottom of each Erlenmeyer flask to form a layer of substrate.

The initial concentration of $NH_4^+$-N for all experiments (Table 1) was determined to be 20.77 mg $l^{-1}$ (10 mg of $[NH_4]_2SO_4$ dissolved in 102.14-ml liquid medium). The composition of the trace element solution used in this study was similar to that of Vallini et al.[12]. The ingredients of each medium, except for $KH_2PO_4$, $NaHCO_3$, and distilled water, were placed into a 250-ml Erlenmeyer flask, and sterilized at 120ºC for 30 min. The $KH_2PO_4$, $NaHCO_3$, and distilled water were sterilized separately and added into the media later. The pH of the media was not adjusted because the $CaCO_3$ powder or coral sands in these media stabilized the pH at approximately 7.0.

**TABLE 1**
**Medium Compositions in the Eight Experiments***

| Exp. No. | Calcium Source | 1.47% $KH_2PO_4$ (µl) | 6.4% $NaHCO_3$ (ml) | Distilled Water (ml) |
|---|---|---|---|---|
| 1 | Coral sands | 140 | 0 | 2 |
| 2 | Coral sands | 140 | 2 | 0 |
| 3 | Coral sands | 0 | 0 | 2.14 |
| 4 | Coral sands | 0 | 2 | 0.14 |
| 5 | $CaCO_3$ | 140 | 0 | 2 |
| 6 | $CaCO_3$ | 140 | 2 | 0 |
| 7 | $CaCO_3$ | 0 | 0 | 2.14 |
| 8 | $CaCO_3$ | 0 | 2 | 0.14 |

* All $CaCO_3$ is in powder. The amount of ammonium sulfate, trace metals, and seawater in each experiment were 10 mg, 50 µl, and 100 ml, respectively.

The experiment factors included the calcium ($Ca^{2+}$) source (Factor A), phosphorus to nitrogen (P:N) molar ratio (Factor B), and carbon to nitrogen (C:N) molar ratio (Factor C) at two levels (see Table 2). Interactions of these factors, i.e., A × B, B × C, and A × C, were also studied. Tests were arranged based on $L_8$ ($2^7$) orthogonal factorial design (Table 3). Concentration of $NO_2^-$-N, not the abundance of AOB in the enrichment media, was selected as the response variable, because of the technical difficulty in counting periphytic AOB via the standard MPN method.

**TABLE 2**
**Experiment Factors and Levels Used in this Study**

| Factor | Level 1 | Level 2 |
|---|---|---|
| A (calcium source) | Coral sands | $CaCO_3$ |
| B (P:N molar ratio) | 1:10 | 0 |
| C (C:N molar ratio) | 0 | 10:1 |

**TABLE 3**
**Arrangement of Factors and Levels in Orthogonal Design***

| | Factor | | | | | | |
|---|---|---|---|---|---|---|---|
| Exp. No. | A | B | A × B | C | A × C | B × C | Blank Column |
| 1 | 1 | 1 | 1 | 1 | 1 | 1 | 1 |
| 2 | 1 | 1 | 1 | 2 | 2 | 2 | 2 |
| 3 | 1 | 2 | 2 | 1 | 1 | 2 | 2 |
| 4 | 1 | 2 | 2 | 2 | 2 | 1 | 1 |
| 5 | 2 | 1 | 2 | 1 | 2 | 1 | 2 |
| 6 | 2 | 1 | 2 | 2 | 1 | 2 | 1 |
| 7 | 2 | 2 | 1 | 1 | 2 | 2 | 1 |
| 8 | 2 | 2 | 1 | 2 | 1 | 1 | 2 |

* Numerical values under Factor denote the level of test. Each experiment had a different medium design. See Table 1 for detail.

## Cultivation

An aliquot of 0.5 ml of well-mixed inoculum was inoculated aseptically into each Erlenmeyer flask containing a specific medium. These were then cultivated in an incubator shaker at 90 rpm under a constant temperature of 28°C in the dark. Samples were withdrawn periodically to determine $NO_2^-$-N production. The experiment was terminated after the final withdrawal at day 5.

## Qualitative Analysis

Soon after the inoculum was added, as well as at day 1, 3, and 5 of incubation, spot tests for $NO_2^-$-N were made. For each spot test, 100 µl of culture medium was withdrawn aseptically from each Erlenmeyer flask and placed into the corresponding alcove on a spot plate. Griess reagents A and B[13] were added to these alcoves sequentially. The color of the culture medium in the spot plate remains unchanged if no $NO_2^-$-N is present, but will change if $NO_2^-$-N is present. The color changes from pink to red and to brown with increases in $NO_2^-$-N concentration.

### Quantitative Analysis

Based on the results from the qualitative tests, quantitative analyses were conducted at day 5 of incubation. Approximately 3 ml of liquid medium were withdrawn from each Erlenmeyer flask and filtered with 0.4 µm *Isopore*@ polycarbonate membranes into aseptic test tubes. The concentration of $NO_2^-$-N in each medium was determined according to standard method[14].

### Data Analysis

The average concentrations of $NO_2^-$-N at Level 1 ($k_1$) and Level 2 ($k_2$) of each factor were calculated. The range of average $NO_2^-$-N concentration, symbolized as R, was calculated as the difference between $k_1$ and $k_2$. A high R value implies a more significant growth response to the culture medium.

## RESULTS

### Qualitative Analysis

Shortly after the enrichment culture was inoculated, the spot test detected AOB activity in each alcove of the spot plates as evidenced by the appearance of a pink color, although no significant difference in color was found among the different culture media. The same results were found at day 3 of inoculation. At day 5 of incubation, however, the spot test detected significant color differences among the treatments (Table 4). Specifically, the media from Flasks 1–4 turned brown, media from Flask 5–7 turned dark red, and media from Flask 8 turned light red. These changes of color were a function of $NO_2^-$-N concentration in the enrichment media, with the highest $NO_2^-$-N concentration in Flask 1–4, followed by Flask 5–7, and Flask 8 (Table 4).

### Quantitative Analysis

The initial concentration of $NH_4^+$-N in each Erlenmeyer flask was 20.77 mg $l^{-1}$. After 5 days of incubation, the average concentration of $NO_2^-$-N in Flask 1, 2, 3, and 4 (containing coral sands) was 11.27 ± 0.64 mg $l^{-1}$, with an average conversion rate of $NH_4^+$-N to $NO_2^-$-N of 54.28 ± 3.08% (Table 4). The average concentration of $NO_2^-$-N in Flask 5, 6, 7, and 8 (containing $CaCO_3$ powder) was 4.94 ± 2.57 mg $l^{-1}$, with an average conversion rate of $NH_4^+$-N to $NO_2^-$-N of 22.09 ± 15.44% (Table 4). The conversion rate in the treatments with coral sands was twice greater than that in the treatments with $CaCO_3$ powder.

The value of R, which measures the difference in average $NO_2^-$-N concentration between Level 1 and 2, was largest for Factor A among all of the treatment interaction (Table 5). Factor A was the most important factor affecting the production of $NO_2^-$-N in our treatments.

**TABLE 4**
**Results from Spot Tests and Nitrite Production on Initial Responses of AOB to Enrichment Medium**

| Exp. No. | Column Response (Color) | | | Nitrite Concentration * (mg $l^{-1}$) |
|---|---|---|---|---|
| | Day 1 | Day 3 | Day 5 | |
| 1 | P | P | B | 10.96 ± 0.02 |
| 2 | P | P | B | 11.35 ± 0.01 |
| 3 | P | P | B | 12.13 ± 0.01 |
| 4 | P | P | B | 10.65 ± 0.02 |
| 5 | P | P | DR | 7.24 ± 0.02 |
| 6 | P | P | DR | 6.27 ± 0.01 |
| 7 | P | P | DR | 4.83 ± 0.03 |
| 8 | P | P | LR | 1.40 ± 0.01 |

*Note:* P = pink; B = brown; DR = dark red; LR = light red.

* Nitrite concentration (mean ± std) was measured at day 5.

**TABLE 5**
**Average $NO_2^-$-N Concentration (mg $l^{-1}$) and Standard Deviation at Level 1 and 2 ($k_1$ and $k_2$) for All of the Experiment Factors at Day 5**

| Level | A | B | A × B | C | A × C | B × C | Blank |
|---|---|---|---|---|---|---|---|
| $k_1$ | 11.27 ± 0.64 | 8.96 ± 2.58 | 7.14 ± 4.85 | 8.79 ± 3.36 | 7.69 ± 4.90 | 7.56 ± 4.44 | 8.18 ± 3.09 |
| $k_2$ | 4.94 ± 2.57 | 7.25 ± 5.02 | 9.07 ± 2.77 | 7.42 ± 4.60 | 8.52 ± 3.04 | 8.65 ± 3.64 | 8.03 ± 4.91 |
| R* | 6.33 | 1.71 | 1.93 | 1.37 | 0.83 | 1.08 | 0.15 |

* R is the difference between $k_1$ and $k_2$.

**TABLE 6**
**Variance Analysis of Experimental Results**

| Source of Variation | SS | df | MS | F |
|---|---|---|---|---|
| A | 80.328 | 1 | 80.328 | 1912.6* |
| B | 5.797 | 1 | 5.797 | 138.0 |
| C | 3.768 | 1 | 3.768 | 89.7 |
| A × B | 7.508 | 1 | 7.508 | 178.7* |
| A × C | 1.370 | 1 | 1.370 | 32.6 |
| B × C | 2.344 | 1 | 2.344 | 55.8 |
| Error | 0.042 | 1 | 0.042 | |

*Note:* $F_{0.05\ (1,1)}$ = 161; * denotes significant difference.

Variance analysis of experimental data (Table 6) shows that the F value for Factor A (Ca source) was greater than $F_{0.05\ (1,1)}$, suggesting that calciumsource had a significant effect on $NO_2^-$-N production.

Because $k_1 > k_2$, Level 1 of Factor A (coral sands) was superior to Level 2 ($CaCO_3$ powder). The F value for interaction A × B was greater than $F_{0.05\,(1,1)}$, but $k_2 > k_1$, suggesting that the interaction of Factor A and B was significant and interactions $A_1B_2$ or $A_2B_1$ provided the best results. In another words, if coral sands were selected as the calcium source, no addition of $KH_2PO_4$ was needed; if $CaCO_3$ powder was selected as the calcium source, $KH_2PO_4$ should be added to attain a molar P:N of 1:10. The F value for Factor C was smaller than $F_{0.05}$, indicating that the two levels of Factor C did not have a differing effect on $NO_2^-$-N production.

## DISCUSSION

### The Roles of Coral Sands in Enrichment Media

Using the change in $NO_2^-$-N concentration as an indicator, this study showed that the growth of AOB was enhanced by the introduction of coral sands into the culture media. The highest $NO_2^-$-N production is associated with coral sands, and without the additions of $CaCO_3$ and phosphate. Possible mechanisms for the higher growth in the enrichment media with coral sands as compared to other media compositions may be twofold. First, the porous coral sands provided more substrate area than the ordinary culture media for AOB, which is periphytic. Second, coral sands served as a nutrient source for the growth of AOB. Coral sands are the remains of the exoskeletons of coral reef animals, which contain many nutrient elements, such as calcium, magnesium, phosphorus, and carbon. When coral sands are introduced, those elements, particularly calcium and phosphorus, can be released into the culture media and used by AOB. This is indirectly indicated by our treatments that showed that $NO_2^-$-N production in the enrichment media with coral sands was higher than with $CaCO_3$ powder, and that no phosphate salt was needed when coral sands were used. However, we did not quantify the release of nutrients from coral sands to the enrichment media. Further studies are needed to understand the relationships between the growth of AOB, surface areas of coral sands, and the nutrient dynamics in the enrichment media.

### The Selection of Culture Media and AOB Diversity

Repeated sequential enrichment of the AOB media may result in a collection of isolates that may not be representative of the biodiversity in the environment[15,16,17,18]. *Nitrosomonas*, which is isolated more commonly than other AOB, does not always dominate in the natural environment[5,17]. Bias selection of enrichment media exists, so the AOB isolated from soil, freshwater, and marine environments could be a very small proportion of the total bacterial population in nature[1]. The compositions of our culture media were similar to the natural conditions inhabited by AOB. We used reduced amount of ammonium sulfate[$(NH_4)_2SO_4$], seawater stored in the dark over a 1-month period as opposed to artificial seawater prepared using distilled water and NaCl. The coral sands are abundant in the coastal marine environment where periphytic AOB are found. However, using porous coral sands in the enrichment of media for AOB could be problematic because the regular calcium source in the enrichment and isolation media of AOB is soluble $CaCl_2$ or insoluble $CaCO_3$ powder, and soluble $CaCl_2$ is used more often than insoluble $CaCO_3$ powder[8,9,16,19,20]. Prior studies indicated that AOB were isolated and purified easily when they were grown in the state of dispersion, so the selection of a liquid medium containing only soluble salts was more likely to occur[8]. Nevertheless, although some AOB such as *Nitrosomonas* grow dispersedly and do not form any type of aggregation[9], others tend to grow on the surface of substrates and/or aggregate into clumps. When coral sands are used in the enrichment media, they benefit the aggregating AOB, which are also very important species in nature.

In the experiment reported here, the inoculum was an enrichment culture, not a pure culture of AOB. When coral sands were used, the amount of $NO_2^-$-N converted from $NH_4^+$-N was higher than when $CaCO_3$ was used. With coral sands as a component of the enrichment media, a more efficient type of AOB for the conversion of ammonium may be selected. In fact, a species of aggregating bacterium that can convert ammonium into nitrite efficiently had been enriched and isolated using the coral sands–containing medium reported in this paper[11].

## Coral Sands and Ammonium-Oxidizing Archaeon (AOA)

The purpose of this study was to enrich and isolate AOB with coral sands as substrates and sources of nutrients. Several recent studies indicate that the ammonia-oxidizing archaeon (AOA) are similar to AOB in many physiological aspects. For example, AOA are chemolithoautotrophical and can use ammonia as the sole source of energy to produce nitrite[21,22]. The first AOA were enriched and isolated from the gravel in a tropical marine tank using a medium that was similar to those media reported for AOB[6]. In addition, the AOA phylotype may be widespread in nitrifying bioreactors, and the mesophilic Archaea were associated with sponges[23,24]. It is possible, therefore, that an enrichment medium containing coral sands, as reported in this paper, is also beneficial for the enrichment of AOA, which also possess a periphytic character.

In summary, this preliminary study demonstrated that, like other porous materials, coral sands can be used in enrichment culture of AOB and the efficiency of $NO_2^-$-N production on this substrate is higher than in enrichment media with $CaCO_2$ powder as the calcium source. No additions of soluble or insoluble calcium and phosphate are needed when coral sands are used. Coral sands are abundant in nature and could potentially play an important role for the enrichments of other nitrifying bacteria.

## ACKNOWLEDGMENTS

This work was supported by Science and Technology Project of Guangdong Province of China (2003C20332 and 2005B20301004). We appreciate comments from Martha Nungesser and Thomas Dreschel on an early draft of this manuscript.
